# Pretreatment platelet-to-lymphocyte ratio (PLR) as a prognosticating indicator for gastric cancer patients receiving immunotherapy

**DOI:** 10.1007/s12672-022-00571-5

**Published:** 2022-11-03

**Authors:** Miaomiao Gou, Yong Zhang

**Affiliations:** 1 Medical Oncology Department, The Fifth Medical Center, Chinese PLA General Hospital, Beijing, People’s Republic of China; 2 Medical Oncology Department, The Second Medical Center, Chinese PLA General Hospital, Fuxing road 28, Haidian district, Beijing, 100853 People’s Republic of China

## Abstract

**Background:**

Previous investigations suggest that systemic inflammation markers are able to provide prognostic value in several cancers. This study seeks to characterize the ability of pretreatment platelet-to-lymphocyte ratio (PLR) to prognosticate advanced or metastatic gastric cancer patients (AGC or MGC, respectively) receiving immunotherapy.

**Methods:**

AGC and MGC patients exposed to PD-1 inhibitors from January 2016–August 2021 in the Chinese PLA General Hospital were recruited. Correlations between PLR and overall survival (OS), progression-free survival (PFS), and immunotherapy-associated tumor response rates were determined.

**Results:**

237 patients were enrolled for this retrospective investigation. The 6 month and 12 month PFS based on the area under the curve value was 0.60 and 0.65 (p < 0.05). based on a calculated PLR cut-off value of 139.41, The PLR < 139.41 group has a longer OS in contrast with the PLR ≥ 139.41 group (13.46 m vs 10.71 m, HR = 0.57, 95% CI 0.42–0.78, p = 0.004). The PLR < 139.41 group had a PFS of 7.93 m in contrast to the 4.75 m seen in those with PLR ≥ 139.41 group (HR = 0.57, 95% CI 0.43–0.76, p = 0.002). The disease control rate (DCR) and objective response rate (ORR) were 86.17% and 30.85%, respectively, in the PLR < 139.41 group, but were 82.52% and 32.17%, respectively in the PLR ≥ 139.41 group. Both groups did not show any marked differences in terms of ORR and DCR (p = 0.887, p = 0.476). PLR is an independent prognostic indicator for OS and PFS upon uni- and multivariate analyses (p < 0.05).

**Conclusions:**

Pre-treatment PLR correlated significantly with PFS and OS in AGC and MGC patients who received immunotherapy. An elevated PLR may provide guidance on subsequent treatment options.

## Background

As the fifth most frequently encountered malignancy, gastric cancer causes 7.7% of all cancer-associated mortality rate (4th highest) [1]. Immunotherapeutic agents including anti-programmed death protein-1 (PD-1) agents have greatly enhanced the treatment options for advanced or metastatic gastric cancer patients (AGC or MGC, respectively). Although immunotherapy combined with conventional chemotherapy confer improved PFS and OS in AGC as reported in the landmark ATTRICATION4 and Checkmate649 studies [2, 3], a considerable patient population fail to respond to an anti-PD-1 based regimen. Several commonly used indices of systemic inflammation, including the neutrophil-to-lymphocyte ratio (NLR) and platelet-to-lymphocyte ratio (PLR) are known for their prognostic value across various cancers [4–8]. Heightened NLR or PLR predict poor prognosis in gastric cancer individuals who received neoadjuvant or chemotherapy [6, 9–11]. A previous study found that NLR levels in AGC patients using third-line nivolumab monotherapy predicted shorter overall survival (OS) [12]. Despite the wealth of knowledge, data is scarce surrounding the relationship between PLR and those treated with anti-pd-1 monotherapy or combined with other agents in AGC or MGC. This study is the first to explore this relationship.

## Patients and methods

AGC or MGC patients were retrospectively enrolled in this observational experiment. Included individuals were those who received immunotherapy between October 1st 2016 and August 31st 2021 at the Chinese PLA General Hospital. Inclusion criteria were: (1) Histopathological confirmation of gastric cancer; (2) Received two minimum of anti-PD-1 treatment cycles; (3) Presence of measurable lesions; (4) Complete availability of clinicodemographic features such as Eastern Cooperative Oncology Group Performance Status (ECOG PS), serum tumor makers, age, gender and surgery history or nutrition status(based on Nutritional risk screening, NRS-2002), an evaluation of tumor response to treatment; (5) A week-in complete blood count prior to any treatment. The exclusion criteria as follow; (1) Patients treated with either cytotoxic lymphocyte antigen 4 (CTLA4) inhibitors or programmed death protein ligand 1 (PD-L1) antibodies were excluded. (2) Received one cycle of anti-PD-1 treatment and no evaluation outcome. (3) insufficient clinical data for analysis. The Ethical Committee of the Chinese PLA General Hospital provided ethical clearance for this observational retrospective study. Written informed consent was obtained from all patients.

### Treatment and assessment of tumor response

The assortment of treatments received by patients in this study encompassed either monotherapy with PD-1 inhibitors, PD-1 inhibitor + chemotherapy, or anti-angiogenic therapy. PD-1–targeting inhibitors used included nivolumab, pembrolizumab, Sintilimab, or toripalimab. Chemotherapy regimens included the SOX (day 1–14 of twice daily S-1 40–60 mg + day 1 oxaliplatin 130 mg/m2 on day 1) or DCF (cisplatin 75 mg/m^2^, docetaxel 75 mg/m^2^ + fluorouracil 750 mg/m^2^/d) and XELOX (day 1–14 of twice daily capecitabine 1000 mg/m^2^ for each cycle and + day 1 intravenous oxaliplatin 130 mg/m^2^ for each cycle) regimens. Anti-angiogenic agents used were either small-molecule tyrosine kinase inhibitors (TKI) (apatinib) or monoclonal antibodies (bevacizumab). Choice of therapy was determined based on patient preference and clinical status.

Tumor degree of response was stratified into either stable disease (SD) or progressive disease (PD), and partial response (PR) or complete response (CR). Disease control was indicated by SD, PR, and CR. CR and PR were also indicative of objective responses. The Evaluation Criteria in Solid Tumors 1.1 assessed tumor response to therapy. Progression-free survival (PFS) was the duration between the time of initiating active therapy until the date of either death or first progression. Overall survival (OS) was the length of time between starting each line of treatment to final follow-up date or death.

### Blood sample analysis

Peripheral blood platelet count and lymphocyte counts taken 1 week-in prior to therapy were recovered from patient records. Division of platelet count with the lymphocyte count yielded the PLR values. Considering the average PFS or OS of late stage gastric cancer is around 6 months or 1 year, the receiver operating characteristic analyses for predicting 6- and 12-month PFS was used to identify an appropriate PLR cutoff value. Patients were then stratified based on this cutoff PLR value.

### Statistical analysis

The SPSS (Windows), version 22 (SPSS Inc., Chicago, IL, USA) was utilized for all data analyses. Median and range were used to describe continuous variables. Categorical data comparisons were done utilizing the Pearson’s chi-square or Fisher’s exact test. The association between PLR and tumor response was assessed using the *χ*^2^ test. The Kaplan–Meier method allowed for survival data analysis. Log-rank analysis was used to contrast survival curves. Cox multivariate was analysis was used to verify independent prognostic parameters. The ideal cut-off value was identified using the Youden Index in ROC analysis.

## Results

**Fig. 1:**
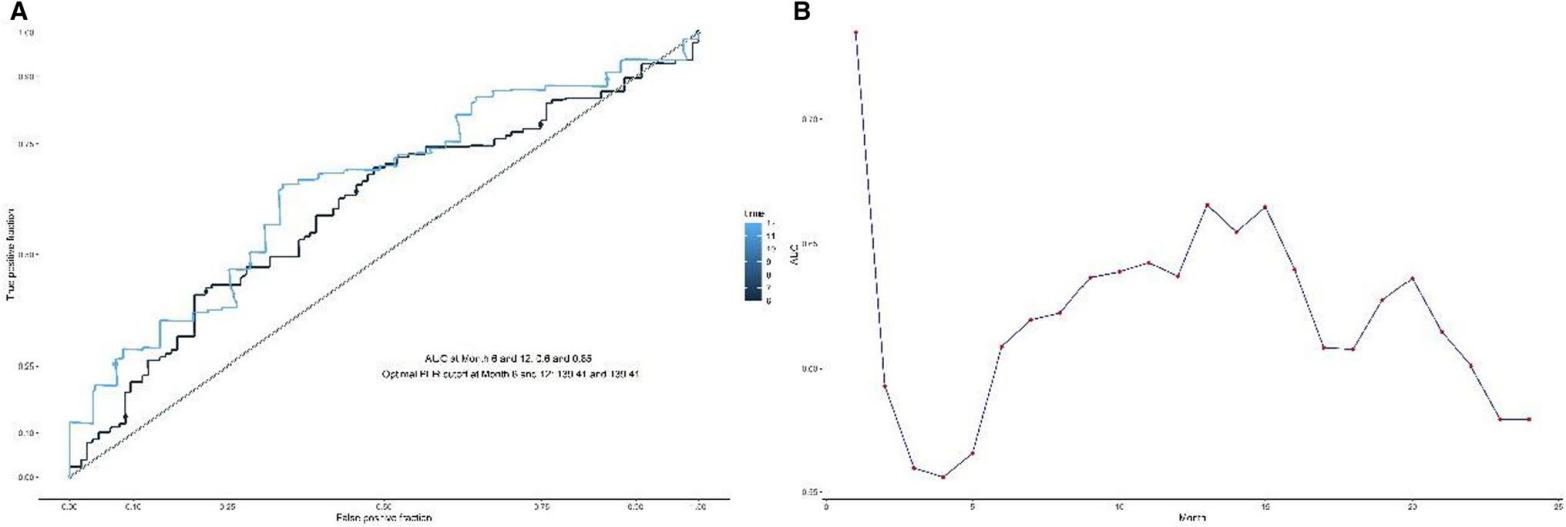
**A** Receiver operating characteristic curves for predicting 6 months and 12 months PFS by the Platelet-to-Lymphocyte Ratio. (AUC area under the curve are 0.60 and 0.65). **B** AUC by PFS time

**Fig. 2:**
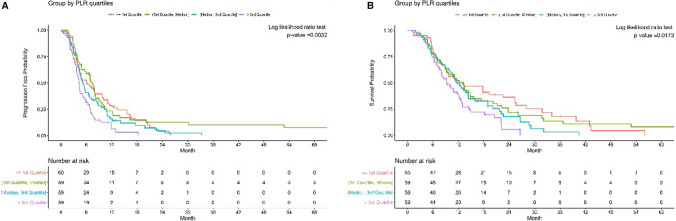
Progression-free survival (PFS) (**A**) and Overall survival (OS) (**B**) in PLR based on quartile

The median age of the 237 enrolled patients was 59 years (range, 34–86) and comprised of 109 males and 128 females. The median OS and PFS were 11.41 m and 5.42 m, respectively. 52.74%patients received first-line immunotherapy. Other patients were treated with first- or second-line chemotherapy alone. 181 patients had received immunotherapy combined with chemotherapy, whereas 56 patients received either immunotherapy monotherapy or combined with anti-angiogenic agents. All patients were of the microsatellite stable (MSS) gastric cancer subtype. 112 patients treated with nivolumab and 57 patients with pembrolizumab, 46 patients with Sintilimab, or 22 patients with toripalimab. The median PLR was 199.2(range, 29.9–1375.6). The areas under curve for PLR at 6- and 12-months were 0.60 and 0.65, respectively based on the most prominent point with a sensitivity of 69.7% and specificity of 66.1% (Fig. 1). Based on these values, the optimal cutoff point for PLR at 6-months and 12 months was 139.41 (p < 0.001). Furthermore, it demonstrated significantly different PFS and OS when patients stratified based on PLR quartile values was (p < 0.05) (Fig. 2).

For the ROC analyses, patients were cohorted into PLR < 139.41 group (low PLR) and PLR ≥ 139.41 group (elevated PLR). There was no significant variability in ECOG-PS, gender, age, number of metastatic sites, PD-L1 expression status, smoking and drinking history, primary tumor site, histological differentiation, surgery history, presence of liver metastasis, carbohydrate antigen 19–9 (CA19-9), carcinoembryonic antigen (CEA), and anti-PD-1 type and treatment line or type, risk of malnutrition (Table 1).Of the 237 patients, 30.85% patients in the PLR < 139.41 group demonstrated an objective response (1 CR and 28 PR). This in contrast to only 32.17% in the PLR ≥ 139.41 group. 86.17% of patients in the low PLR group demonstrated confirmed disease control rate (DCR), while only 82.52% achieved this status in the elevated PLR group. The two groups demonstrated no differences in terms of ORR and DCR (p = 0.887, p = 0.476) (Table 2).

**Table 1:** The characteristics of the analyzed patients in different groups

Characteristics	PLR < 139.41		PLR ≥ 139.41		p value
	N	%	N	%	
No. patients	94		143		
Gender, n (%)					0.184
Male	38	40.43	71	49.65	
Female	56	59.57	72	50.35	
Age median = 59, n (%)					0.409
< 59	31	32.98	56	39.16	
≥ 59	63	67.02	87	60.84	
PD-L1 n (%)					0.396
Positive (CPS > 1%)	44	46.81	63	44.06	
Negative (CPS < 1%)	29	30.85	37	25.87	
Unknown	21	22.34	43	30.07	
ECOG PS, n (%)					0.415
0	30	31.91	35	24.48	
1	47	50.00	76	53.15	
≥ 2	17	18.09	32	22.38	
Tumor_location, n (%)					0.509
Cardia	27	28.72	32	22.38	
Body/Fundus	56	59.57	95	66.43	
Pylorus	11	11.70	16	11.19	
Histological_differentiation, n (%)					0.591
Poorly	47	50.00	81	56.64	
Moderately	43	45.74	56	39.16	
Well	4	4.26	6	4.20	
Surgery history					0.186
Yes	49	52.2	62	43.3	
NO	45	47.8	81	56.7	
No. of metastasis organs, n (%)					0.598
< 2	52	55.32	74	51.75	
≥ 2	42	44.68	69	48.25	
Liver metastasis, n (%)					0.344
Yes	40	42.55	52	36.36	
No	54	57.45	91	63.64	
Smoking history					0.566
Yes	41	43.62	57	39.86	
No	53	56.38	86	60.14	
Drinking history					0.602
Yes	52	55.32	84	58.74	
No	42	44.68	59	41.26	
Risk of malnutrition					0.151
1–2	67	71.3	89	62.2	
3–4	27	28.7	54	37.8	
CEA, n (%)					0.507
< 5 ng/ml	45	47.87	76	53.15	
≥ 5 ng/ml	49	52.13	67	46.85	
CA199, n (%)					0.424
< 37 U/mL	50	53.19	84	58.74	
≥ 37 U/mL	44	46.81	59	41.26	
PD-1 inhibitor					0.939
Nivolumab	46	48.93	66	46.15	
Pembrolizumab	20	21.27	37	25.87	
Sintilimab	18	19.14	28	19.58	
Toripalimab	10	10.64	12	8.39	
Anti-pd-1 treatment line n (%)					0.676
First line	52	55.32	73	51.05	
Second line	35	37.23	55	38.46	
Third line	7	7.45	15	10.49	
Anti-pd-1 treatment type n (%)					0.717
Anti-pd-1 monotherapy	5	5.32	11	7.69	
Combination therapy					
Anti-pd-1 plus chemotherapy	74	78.72	107	74.83	
Anti-pd-1 plus anti-angiogenic therapy	15	15.96	25	17.48

**Table 2:** Treatment response in the PLR < 139.41 group and in the PLR  ≥ 139.41 group

Response	PLR < 139.41		PLR ≥ 139.41		P value
	N	%	N	%	
No. patients	94		143		
Response					
CR	1	1.06	2	1.40	
PR	28	29.79	44	30.77	
SD	52	55.32	72	50.35	
PD	13	13.83	25	17.48	
ORR					0.887
CR + PR		30.85		32.17	
DCR					0.476
CR + PR + SD		86.17		82.52	

**Fig. 3:**
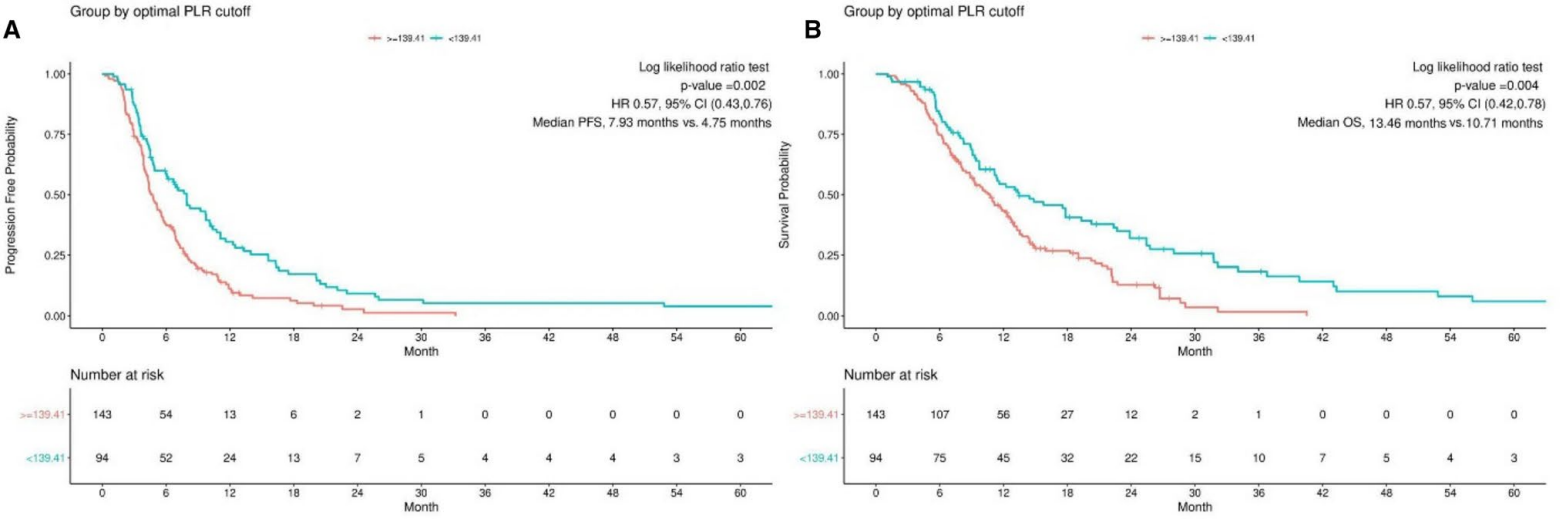
Progression-free survival (PFS) (**A**) and Overall survival (OS) (**B**) in PLR based on the PLR cut-off value

Univariate analyses allowed for identification of clinical factors which correlated to PFS or OS. Patients with raised PLR had a median PFS of 4.75 m in contrast to the 7.93 m seen in those with low PLR (HR = 0.57, 95% CI 0.43–0.76, p = 0.002) (Fig. 3). Those with low PLR had a longer median OS than those with elevated PLR (13.46 m vs 10.71 m, HR = 0.57, 95% CI 0.42–0.78, p = 0.004) (Fig. 3). Univariate analysis revealed that those patients with low PLR group or receiving anti-pd-1 plus chemotherapy had better OS than patients with elevated PLR or receiving immunotherapy monotherapy or combined with antigenic agents (P < 0.05) (Table 3).However, multivariate analysis found only PLR to be an independent prognostic biomarker for PFS (HR = 0.58, 95%CI,0.43 -0.78, P < 0.001) (Table 4). PLR and antipd-1 treatment type,surgery history were three independent prognostic factors for OS upon multivariate analysis (HR = 0.59, 95% CI 0.43–0.82, P = 0.001, HR = 1.92, 95% CI 1.29–2.85, P = 0.001; HR = 1.53, 95% CI 1.11–2.11, P = 0.009, respectively) (Table 3). We therefore conclude that elevated PLR was associated with inferior PFS and OS.

**Table 3:** Univariate analysis and multivariate analysis of overall survival time (OS)

Variable category	Category	Univariate analysis	Multivariate analysis	
		p-value	HR (95% CI)	p-value
Gender	Female versus male	0.448	1.19 (0.87–1.63)	0.261
Age	≥ 59 versus < 59	0.878	1.00 (0.72–1.40)	0.968
ECOG PS	≥ 2 versus 0–1	0.694	0.82 (0.56–1.18)	0.291
Tumor location	Pylorus versus body/fundus versus cardia	0.406	1.08 (0.82–1.41)	0.568
Histological differentiation	Poorly versus moderately and well	0.984	0.94 (0.69–1.27)	0.701
Surgery	No verse yes	0.014	1.53 (1.11–2.11)	0.009
No.of metastasis organs	≥ 2 verse = 1	0.561	0.95 (0.69–1.30)	0.765
Liver metastasis	No verse yes	0.236	1.18 (0.85–1.65)	0.300
Smoking history	No verse yes	0.154	1.26 (0.91–1.75)	0.153
Drinking history	No verse yes	0.926	1.07 (0.78–1.47)	0.671
PD-1 Inhibitor	Nivolumab verse Pembrolizumab verse Sintilimab verse Toripalimab	0.734	0.98 (0.84–1.15)	0.845
Anti-pd-1 treatment line	Second or more line verse first line	0.288	0.92 (0.65–1.31)	0.665
Anti-pd-1 treatment type	Anti-pd-1 monotherapy or antiagenis verse anti-pd-1 plus chemotherapy	0.001	1.92 (1.29–2.85)	0.001
PLR	< 139.41 versus ≥ 139.41	0.004	0.59 (0.43–0.82)	0.001

*CI* confidence interval; *ECOG PS* Eastern Cooperative Oncology Group Performance Status; *PLR* platelet to lymphocyte ratio; *HR* hazard ratio

**Table 4:** Univariate analysis and multivariate analysis of progression free time (PFS)

Variable category	Category	Univariate analysis	Multivariate analysis	
		p-value	HR (95% CI)	p-value
Gender	Female versus male	0.628	1.00 (0.74–1.34)	0.997
Age	≥ 59 versus < 59	0.466	0.86 (0.633–1.17)	0.354
ECOG PS	≥ 2 versus 0–1	0.063	1.29 (0.92–1.82)	0.136
Tumor location	Pylorus versus body/fundus versus cardia	0.328	1.04 (0.81–1.34)	0.716
Histological differentiation	Poorly versus moderately and well	0.681	1.06 (0.79–1.41)	0.676
Surgery	No verse yes	0.095	1.21 (0.90–1.63)	0.196
No.of metastasis organs	≥ 2 verse = 1	0.877	0.93 (0.69–1.25)	0.650
Liver metastasis	No verse yes	0.740	0.94 (0.69–1.27)	0.714
Smoking history	No verse yes	0.321	1.07 (0.79–1.44)	0.660
Drinking history	No verse yes	0.155	0.78 (0.58–1.05)	0.105
PD-1 Inhibitor	Nivolumab verse Pembrolizumab verse Sintilimab verse Toripalimab	0.742	1.02 (0.89–1.18)	0.702
Anti-pd-1 treatment line	Second or more line verse first line	0.354	1.01 (0.73–1.39)	0.947
Anti-pd-1 treatment type	Anti-pd-1 monotherapy or antiagenis verse anti-pd-1 plus chemotherapy	0.137	1.278 (0.88–1.85)	0.195
PLR	< 139.41 versus ≥ 139.41	0.002	0.58 (0.43–0.78)	0.000

*CI* confidence interval; *ECOG PS* Eastern Cooperative Oncology Group Performance Status; *PLR* platelet to lymphocyte ratio; *HR* hazard ratio

## Discussion

Immunotherapy has been popularized recently in the field of AGC and MGC treatment. Several experiments have researched the ability of routine blood parameters in prognosticating AGC and MGC patients treated with immunotherapy. Parameters such as microsatellite instability, tumor mutation burden (TMB), PD-L1 expression, and tumor infiltrating lymphocyte (TIL) counts have already been identified as markers with likely prognostic value in patients with cancer [13–17]. However, these biomarkers are not commonly available in clinical laboratories, and are time-consuming, require technical expertise, and incur significant cost. Some of these parameters are only available after resection of the primary tumor, which is not feasible in some cases. Therefore, reliable and conveniently available prognostic factors are crucial in guiding patient management.

Systemic inflammation indices such as NLR, PLR, and MLR have previously been affirmed to be able to predict OS in several types of cancers [9, 18–22]. PLR may be a vital player in prognosticating AGC and MGC [11, 23]. However, whether or not PLR holds the same prognostic ability in AGC and MGC patients treated with immunotherapy is not clear. Our study is the first that provides concrete evidence linking PLR to poor OS in this cohort, suggesting that clinicians should be cautious in initiating immunotherapy especially in these patients. Kaplan–Meier survival analysis revealed curves for PFS and OS in patients with pre-treatment PLR < 139.41and PLR ≥ 139.41 that were significantly different. Multivariate analysis further underscored the predictive value of PLR both in PFS and OS. This finding was consistent with other studies in patients treated with chemotherapy [24]. The pathophysiology of this phenomenon has yet to be characterized. We postulate that high PLR correlates to poor OS given that platelet activation features consistently in all steps of tumorigenesis starting from tumor initiation, spread, and metastasis [25]. Additionally, some study showed that platelet also promote tumor metastasis and angiogenesis by releasing various growth factors such as vascular endothelial growth factor-A. The platelet formed can also promote tumor cell immune escape and resistance to chemotherapeutic drugs [24]. Furthermore, lower OS has been observed in cancer patients with thrombocytosis [26]. Decreased T cells on the other hand, is known to indicate improved prognosis across several different cancers. This has been attributed to the ability of these immune regulating cells that hides tumor cells from immune surveillance [27]. Likewise, increased TIL is also linked to increased responsiveness to immune checkpoint therapies [28]. Raised PLR ratios are therefore indicative of a cellular milleu that is highly conducive for tumor growth and poor response to immunotherapy. It is unsurprising that an elevated PLR corresponds to inferior survival due to a high proportion of tumor-promoting platelets and reduction of tumor-killer lymphocytes.

However, our study did not demonstrate a significant ability of PLR for predicting the response rate in AGC or MGC patients treated with PD-1 inhibitors. Similarly, other studies have instead shown a correlation between PLR and chemotherapeutic response [6, 23]. The inconsistencies in results are unclear. It is assumed that chemotherapy exerts toxicity directly on tumor cells while immunotherapy induces a long-term, gradual chemotoxic environment as PFS KM-Curves of keynote177 showed us [29].

Our investigation revealed a calculated cut-off PLR value of 139.41 with an ROC curve based on the 6 or12month PFS. The cut-off PLR level was different compared with previous studies. Jin Wang et al. who also assessed the relationship between PLR and response and survival of patients receiving first-line chemotherapy used a PLR cut-off of 201.6 [23]. The highest PLR cut-off level from a meta-analysis based on 28 studies was at level of 350 [24]. These differences may be attributed to different laboratory reference standards.

Our study is limited by the fact that it is a retrospective study with a small sample size. Furthermore, the values of other inflammatory markers such as NLR and MLR were not calculated. In order for these results to be validated, studies on larger cohorts are necessary. And also, different cutoff value for PLR which might influence the result of study. Lastly, another limitation is variation of treatment regimen and immune checkpoint inhibitors line which can have a significant impact on the outcome of this study.

## Conclusion

Pre-treatment PLR has prognostic value in AGC and MGC patients treated with immunotherapy. This information is useful in aiding selection of therapy for individuals with gastric cancer.

## Data Availability

The datasets used and/or analyzed during the current study are available from the corresponding author upon reasonable request.
